# Eukaryotic rpL10 drives ribosomal rotation

**DOI:** 10.1093/nar/gkt1107

**Published:** 2013-11-08

**Authors:** Sergey O. Sulima, Suna P. Gülay, Margarida Anjos, Stephanie Patchett, Arturas Meskauskas, Arlen W. Johnson, Jonathan D. Dinman

**Affiliations:** ^1^Department of Cell Biology and Molecular Genetics, University of Maryland, College Park, MD 20742, USA, ^2^Section of Molecular Genetics and Microbiology, Institute for Cellular and Molecular Biology, University of Texas at Austin, Austin, TX 78712, USA and ^3^Department of Biotechnology and Microbiology, Vilnius University, Vilnius LT-03101, Lithuania

## Abstract

Ribosomes transit between two conformational states, non-rotated and rotated, through the elongation cycle. Here, we present evidence that an internal loop in the essential yeast ribosomal protein rpL10 is a central controller of this process. Mutations in this loop promote opposing effects on the natural equilibrium between these two extreme conformational states. rRNA chemical modification analyses reveals allosteric interactions involved in coordinating intersubunit rotation originating from rpL10 in the core of the large subunit (LSU) through both subunits, linking all the functional centers of the ribosome. Mutations promoting rotational disequilibria showed catalytic, biochemical and translational fidelity defects. An rpL3 mutation promoting opposing structural and biochemical effects, suppressed an rpL10 mutant, re-establishing rotational equilibrium. The rpL10 loop is also involved in Sdo1p recruitment, suggesting that rotational status is important for ensuring late-stage maturation of the LSU, supporting a model in which pre-60S subunits undergo a ‘test drive’ before final maturation.

## INTRODUCTION

The ribosome is an essential and complex nanomachine that provides a model for understanding principles of macromolecular assembly and functional coordination. The eukaryotic yeast ribosome is a 3.6-MDa RNA–protein complex, consisting of 79 intrinsic ribosomal proteins and 4 ribosomal RNAs (rRNAs) (1). Different biochemical functions are spatially separated from one another in the two subunits of the ribosome. The small subunit (SSU) contains the mRNA decoding center, whereas the large subunit (LSU) harbors separate regions with distinct functions, the peptidyltransferase center (PTC) (responsible for catalysis), the peptide exit tunnel, three transfer RNA (tRNA) binding pockets and a single binding site that must distinguish between the two elongation factors and release factor in response to specific circumstances. The subunits must also interact with one another as a holoenzyme to coordinate a complex series of events, particularly allosteric movements that occur throughout the course of the elongation cycle. The two extreme conformational states are termed ‘rotated’ (also known as ratcheted or hybrid) and ‘non-rotated’ (also known as classical). How information is exchanged over long distances between spatially distinct functional centers, and how these centers then work in concert to ensure timely rotation, proper ligand binding, and ultimately unidirectional and faithful translation remains largely unclear. In addition, while the roles of the SSU (particularly the head) and tRNAs in ribosome rotation have been investigated, the question of whether the LSU is an active or passive participant in this process has not been explored.

**Figure 1.**
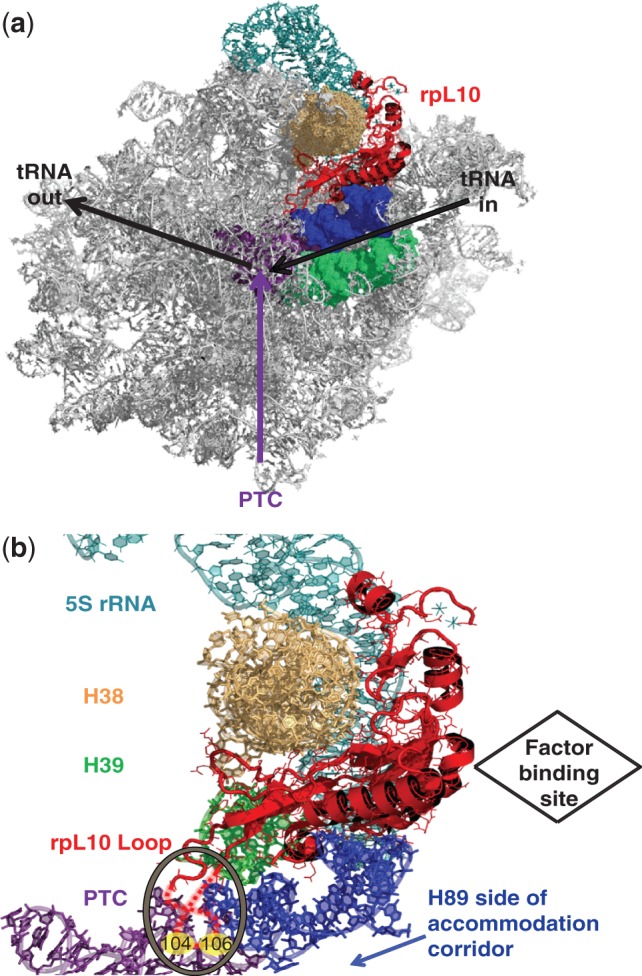
rpL10 is strategically positioned in the core of the LSU. **(a)** The big picture: rpL10 in the context of the subunit interface of the LSU. **(b)** A close-up view of rpL10 and the local environment. The hypothetical loop structure is circled and indicated by dashed red lines, and the approximate positions of S104 and A106 are indicated. The protein is situated between Helices 38 and 89, and appears to be an extension of Helix 39. It is located in close proximity to several functional centers of the LSU including the PTC, the aa-tRNA AC and the elongation factor binding site. It is also positioned to communicate with the SSU through Helix 38 and the 5S rRNA. Images were generated using PyMOL.

Ribosomal protein L10 [rpL10, aka L16 (1)] plays essential roles in ribosome biogenesis and translational fidelity. Incorporation of rpL10 into the LSU in the cytoplasm (2) constitutes a late step of LSU maturation. rpL10 works in conjunction with Shwachman-Diamond protein Sdo1p and the eukaryotic elongation factor 2 (eEF2)-like GTPase Efl1p to promote the release of the anti-association factor Tif6p (3–5) and the nuclear export adapter Nmd3p (6). Thus, LSUs lacking rpL10 are unable to join with the SSU (5). rpL10 is located near the corridor through which aminoacyl-tRNAs (aa-tRNAs) move during the process of accommodation (Figure 1a) and is involved in tRNA movement through this structure (7). It is also located near several other functional centers of the LSU, including the PTC, the A-site finger (H38), the elongation factor binding site and the GTPase associated center (Figure 1b). The C-terminus of rpL10 contacts 5S rRNA, which interacts with rpL5 and rpL11 at the head of the central protuberance (8). Thus, rpL10 is well positioned to act as a sensor of activity near the PTC, and transduce that information to other functional centers to coordinate ribosome function.

An essential internal loop of rpL10 (aa 102–112, previously called the ‘P-site loop’) that makes the closest approach to the PTC (13 Å) of any ribosomal protein is crucial for Tif6p and Nmd3p release (5,9). Mutagenesis of the loop revealed two classes of mutants based on their effects on ribosome biogenesis (5). This study employs the strongest representative mutants of these two classes: S104D (improper subunit joining and 60S biogenesis defect) and A106R (high 60S/40S ratio). Here, we present evidence that these mutations promote opposing effects on the natural equilibrium between the two extreme conformational states of the ribosome. Structural and biochemical analyses of vacant ribosomes demonstrate that the S104D mutation drives the ribosome toward the rotated state, favoring binding of eEF2 and disfavoring binding of eEF1A/aa-tRNA/GTP (elongation ternary complex) and inhibiting peptidyltransfer. In contrast, A106R causes vacant ribosomes to distribute toward the non-rotated state, favoring elongation ternary complex binding over eEF2 and leaving peptidyltransferase activity unaffected. We also show that the non-rotated conformation favors Sdo1p binding, stimulating binding of tRNAs to the A-site. Furthermore, Sdo1p competes with acetylated-aa-tRNA for binding to the P-site and inhibits peptidyltransfer, suggesting that Sdo1p stabilizes the non-rotated state through binding the P-site. Large scale rRNA chemical modification analyses reveal distinct information transmission pathways originating from rpL10 in the heart of the LSU and emanating throughout the LSU and the SSU.

These observations lead us to propose that the rpL10 loop plays a central role in much of the ribosomal lifecycle by helping to set the conformational status of the LSU, coordinate intersubunit rotation and communicate this information to the decoding center on the SSU. During late LSU biogenesis, the loop senses Sdo1p recruitment to the P-site, initiating a ‘test drive’ to ensure the functionality of pre-60S subunits. After ensuring proper 80S assembly, the loop monitors the tRNA occupancy status of the PTC A-site: in the absence of A-site ligand (aa-tRNA) it can sample this space, while the introduction of ligand displaces it. We suggest that the positioning of the rpL10 loop determines which state the LSU assumes in a process involving cascades of allosteric interactions that link functional centers in the LSU with those in the SSU. The downstream effects of rotational disequilibrium are wide-ranging, impacting translational reading frame maintenance, the ability to discriminate between cognate and near- and non-cognate codons and termination codon recognition. Mutants of ribosomal protein L3 that confer opposing effects on ribosome structure and function can suppress the structural, biochemical and functional defects of the rpL10 loop mutants by re-establishing the normal rotational equilibrium. In sum, we propose that the rpL10 loop is a master controller of ribosome structure and function, influencing critical steps in both ribosome assembly and biogenesis and the protein-synthetic phase of elongation. We suggest that the unidirectionality of translation is aided by this intrinsic feature of the ribosome, and that the LSU alone has the ability to independently influence intersubunit rotation.

## MATERIALS AND METHODS

### Strains, plasmids and genetic manipulation

*Saccharomyces cerevisiae* strain AJY1437 containing wild-type *RPL10* on a centromeric *URA3* vector (pAJ392) has previously been described (5). In AJY3222, the wild-type vector was replaced by wild-type *RPL10* on a centromeric *LEU2* vector (pAJ2522) through standard 5-FOA shuffling techniques. AJY3209 harbors pAJ2609, a centromeric *LEU2* vector expressing the *rpl10-S104D* allele. Similarly, AJY3212 contains pAJ2612, which expresses the *rpl10-A106R* mutant from a centromeric *LEU2* vector. Generation of the *rpl10-F94I* and *rpl10-G81D* expressing strains, JD1308.F94I and JD1308.G81D, was previously described (7). AJY2104 (9) was transformed with pAJ2522 or pAJ2609 and *RPL3* or *rpl3-W255C* expressed from 2 µm *TRP1* vectors. Standard molecular biology techniques were used to subclone *RPL3* and *rpl3-W255C* into the *HIS3* selectable pRS423. AJY3209 was subsequently transformed with pRS423-*RPL3* and pRS423-*rpl3-W255C*.

### Translational fidelity and polysome analyses

The dual luciferase reporter plasmids pYDL-control, pYDL-LA, pYDL-Ty*1*, pYDL-UAA (10), pYDL-AGC_218_ and pYDL-TCT_218_ (11) were employed to monitor programmed −1 ribosomal frameshifting, programmed +1 ribosomal frameshifting, suppression of UAA, and suppression of an AGC near-cognate serine codon and a TCT non-cognate serine codon in place of the cognate AGA codon in the firefly luciferase catalytic site, respectively. The reporters were expressed from high-copy *URA3*-based plasmids (pJD375, pJD376, pJD376, pJD431, pJD642 and pJD643). Assays were performed as previously described (12). Sample readings were collected using a GloMax Multi-Microplate luminometer (Promega). All assays were repeated four times. Sucrose density gradient analysis was carried out as described (5).

### Ribosome preparation

Purification of active 80S ribosomes using cysteine-charged sulfolink columns was performed as described (13), with the following modifications: after elution from the column, ribosomes were treated with 1 mM final concentration GTP and 1 mM final concentration pH-neutralized puromycin at 30°C for 30 min to remove endogenous tRNAs. After a 100 000 × g 16–20 h spin through a high salt glycerol cushion [20 mM HEPES-KOH pH 7.6, 60 mM NH_4_Cl, 500 mM KCl, 10 mM Mg(OAc)_2_, 2 mM DTT, 25% glycerol], ribosomes were resuspended in elution buffer and passed through a low salt cushion [20 mM HEPES-KOH pH 7.6, 50 mM NH_4_Cl, 5 mM Mg(OAc)_2_, 1 mM DTT, 25% glycerol] to increase purity.

### Ribosome/tRNA interactions

eEF1A preparation, purification of aa-tRNA synthetases, charging of tRNA^Phe^ with [^14^C]-phenylalanine and purification of aa-tRNA and acetylated aa-tRNA were carried out as described (14,15). To assay steady-state dissociation rates (*K*_D_) of aa-tRNA to the ribosomal A-site, two sets of reactions were set up in parallel. A mix containing 100 µg of polyU, 50 pmol of ribosomes, a 4-fold molar excess of tRNA^Phe^, all in binding buffer [80 mM Tris–HCl, pH 7.4 at 30°C, 160 mM NH_4_Cl, 15 mM Mg(CH_3_COOH)_2_, 2 mM spermidine, 0.5 mM spermine, 6 mM β-mercaptoethanol] in 150 µl total volume was prepared and incubated for 30 min at 30°C to block the P-site. To prepare the ternary complex ([^14^C]Phe-tRNA^Phe^•eEF1A•GTP), 100 µg of soluble protein factors, 1 mM final concentration of GTP, 125 pmol [^14^C]-Phe-tRNA^Phe^ were mixed in 50 µl total volume binding buffer and incubated for 30 min at 30°C. After incubation, serial 2-fold dilutions of the ternary complex reaction mix were prepared, resulting in eight fractions containing decreasing amounts of ternary complex (62.5–1 pmol), in 105 µl each. An equal amount of the ribosome mix (5 pmol of ribosomes, 15 µl) was added to each dilution, followed by incubation for 30 min at 30°C. The mixtures were applied onto pre-wetted nitrocellulose Millipore HA (0.45 µm) filters, washed with binding buffer, and radioactivity was measured via scintillation counting. Background control reactions without ribosomes were performed at each ligand dilution and subtracted from experimental ones. Binding data were analyzed via single binding site with ligand depletion models using GraphPad Prism. Non-enzymatic binding studies were performed likewise, but without eEF1A or by using 50 µg of soluble protein factors (half maximum activity). To test binding of tRNA to the P-site, Ac-[^14^C]-Phe-tRNA^Phe^ was used as the ligand, there was no need to pre-block with tRNA^Phe^ and 11 mM Mg(CH_3_COOH)_2_ was used in the binding buffer. All reactions were repeated four times.

### Peptidyltransferase activity

Single turnover peptidylpuromycin reactions were performed to assay apparent rates of peptidyltransfer as described (14) with the following modification. Ribosomes pre-bound with Ac-[^14^C]Phe-tRNA^Phe^ and polyU were loaded onto pre-wetted Millipore HA (0.45 µm) filters and washed with binding buffer. The filters were placed into 15 ml scintillation vials and 2.4 ml of binding buffer + 0.05% Zwittergent (EMD BioScience), followed by a 30 min incubation on a rocker at 4°C. Aliquots (1 ml) were taken from each vial, incubated for 5 min at 30°C to activate ribosomes, reactions were initiated by adding pH-neutralized puromycin to 10 mM final concentrations, and the procedure completed as described (14). Reactions were repeated four times.

### Sdo1p cloning, purification and labeling

Sdo1p was cloned into a modified pET-21 a expressing Sdo1p with a C-terminal 6× histidine tag and the phosphorylatable kemptide. The protein was expressed in Codon Plus bacteria (Stratagene) and purified by Ni-NTA (Invitrogen) chromatography followed by gel filtration on sephacryl S200 (GE Healthcare). Labeling reactions containing 10 µg of Sdo1p, 5× molar excess of [^32^P]-γ-ATP, 1 µl PKA (NEB) in 100 µl 1× kinase buffer (NEB) were incubated for 15 min at 30°C, passed through G25 columns (GE) to remove unincorporated ATP, and flow-through was measured by scintillation counting. Flow-through values from control mixtures lacking PKA were subtracted from values of the labeling reactions to determine the specific activity of ^32^P-labeled Sdo1p, yielding 40–60% label incorporation.

### Ribosome/protein interactions

6×-His-tagged eEF2 was purified from TKY675 yeast cells (a generous gift from Dr T. Kinzy) as described (16). Aliquots containing ribosomes (2 pmol) were first pre-incubated with increasing concentrations of eEF2 (0.25–32 pmol), 100 µg of polyU, 0.1 mM final concentration GDPNP and 4× molar excess of [^14^C] NAD over ribosomes in 50 µl total volume binding buffer at 30°C for 20 min. Diphtheria toxin (0.2 µg) was added, and reactions were incubated for 30 min at 30°C. After precipitation with TCA (final concentration 15%) and 15 min incubation on ice, reaction mixtures were applied onto GF/C filters, washed with 5% TCA, and the amount of [^14^C]-ADP ribosylated eEF2 was determined by scintillation counting. Readings reflect unbound eEF2 and were subtracted from total eEF2 to obtain values bound. Sdo1p binding assays were performed by incubating ribosomes (2 pmol) with increasing concentrations of Sdo1p (0.25–32 pmol) and 100 µg of polyU in binding buffer [50 mM Tris-(OAc)_2_ pH 7.5 RT, 50 mM NH4(OAc)_2_, 10 mM Mg(OAc)_2_, 2 mM DTT] in 50 µl total volume at 30°C for 20 min. The reaction mixtures were then applied onto pre-equilibrated 1 ml polyethylene filter spin columns (Pierce) containing 0.3 ml cysteine-charged sulfolink resin and incubated on ice for 5 min. The columns were spun and flow-through measured via scintillation counting. Readings reflect unbound Sdo1p and were subtracted from total loaded to obtain Sdo1p bound. Background control reactions without ribosomes were performed at each ligand dilution. eEF2 assays were repeated four times, Sdo1p assays were performed in triplicate.

### Ribosome binding competition

To monitor effects of Sdo1p on tRNA binding to the A-site, wild-type ribosomes (270 pmol) primed with polyU (0.35 mg), were first mixed with a 4-fold molar excess of uncharged tRNA^Phe^, 2.8 nmol GTP, and 5 µg of soluble protein factors (including eEF1A) in 700 µl total volume of ribosome binding buffer. After 10 min incubation at 30°C, ribosome/tRNA complexes (50 µl aliquots) were added to 10 µl aliquots of 2-fold dilutions of purified Sdo1p (500–31 pmol, plus a no-Sdo1p control) and incubated at 30°C for 10 min. Subsequently, 25 pmol of [^14^C]Phe-tRNA was added to each ribosome/Sdo1p complex, and incubated at 30°C for 10 min. The reaction mixtures were applied onto pre-wetted nitrocellulose Millipore HA (0.45 µm) filters, washed with binding buffer, and radioactivity was measured via scintillation counting. Background control reaction values from samples without ribosomes were subtracted from experimental ones. To monitor the ability of Sdo1p to compete for binding at the P-site, the same conditions were employed except that binding reactions were performed in 11 mM magnesium, and [^14^C]Ac-Phe-tRNA^Phe^ was used. Assays of peptidyltransferase activity were performed as described above in the presence of Sdo1p. All assays were performed twice in triplicate.

### Preparation of complexes for hSHAPE probing

Fifty pmoles 80S ribosomes isolated from isogenic strains were incubated with 100 µg polyuridylic acid (polyU) in binding buffer [80 mM HEPES pH 7.5, 50 mM NaCl, 11 mM Mg(OAc)_2_, 6 mM β-mercaptoethanol] at 30°C for 10 min. One set of ribosomes so prepared (‘vacant 80S’ wild type, rpL10-S104D, rpL10-A106R) was employed for chemical protection assays. To prepare control ‘non-rotated’ ribosomes, 200 pmol *N*-acetyl-phenylalanyl tRNA^Phe^ were added and incubations continued for 20 min. To prepare ‘rotated’ wild-type ribosomes, vacant 80S ribosomes were first incubated with 200 pmol of deacylated tRNA^Phe^. eEF2 (400 pmol) and GDPNP (final concentration of 1 mM, Sigma) were then added to the deacylated tRNA^Phe^—ribosome mixture and incubated for an additional 20 min. These conditions were based on results of binding assays.

### rRNA structure probing

hSHAPE (17) of rRNA with 1M7 was performed as described (18) using the following substrates: vacant ribosomes isolated from isogenic wild-type cells, cells expressing *rpl10-S104D*, *rpl10-A106R*, wild-type ribosomes containing acetylated-aa-PhetRNA^Phe^ in the P-site (non-rotated controls) and deacylated-tRNA^Phe^ + eEF2-GDPNP (rotated control). The following primers were employed: 969 and 1780 in the SSU; 25-2, 1466, 2632, 2836, 25-7 and 3225 in the LSU. Data were analyzed using SHAPEFinder (19). For kethoxal studies, 25 pmol of ribosomes in a 50 -µl volume were treated with 1 µl of a 4% kethoxal solution (in pure ethanol), or 1 µl of ethanol as control, and incubated for 10 min at 30°C. Reactions were stopped by addition of one half volume of stop solution (150 mM sodium acetate, 250 mM potassium borate), followed by analysis as above using primer 969.

### Statistical analyses

Student’s *t*-test for *P*-value calculations was used throughout. Data analysis of dual-luciferase assays and rRNA probing was carried out as described (12,18). After generation of rRNA chemical modification data using ShapeFinder (19), the median reactivities of each primer region were used to normalize the raw integrated peak values.

## RESULTS

### The S104D and A106R mutants promote opposing effects on ligand binding to the ribosomal A- and P-sites

**Figure 2.**
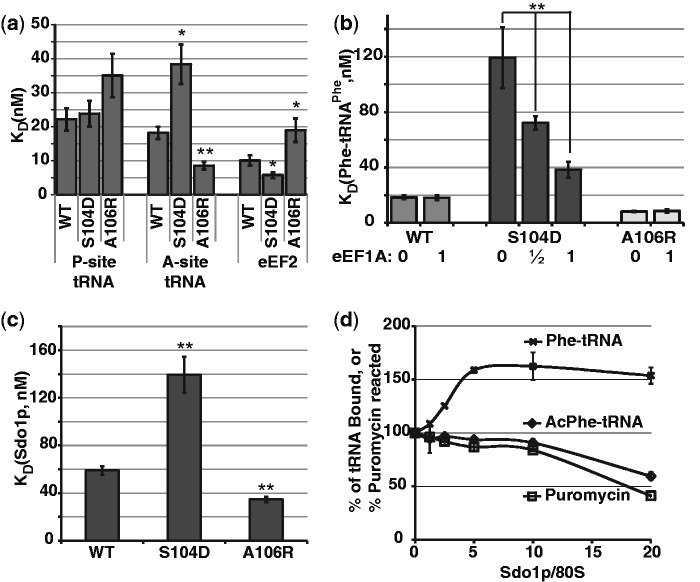
rpL10 loop mutants inversely affect ligand binding to the A- and P-sites. **(a–c)** Steady state binding of indicated ligands for ribosomes. (a) Dissociation constants obtained from assays of binding of Ac-aa-tRNA to the P-site, aa-tRNA to the A-site and eEF2 to ribosomes isolated from cells expressing wild-type rpL10 and the rpL10-S104D and rpL10-A106R mutants. (b) Titration of eEF1A into aa-tRNA binding reactions similar to (a). (c) Dissociation constants obtained from assays of binding of Sdo1p to indicated ribosomes. **(d)** Competition assays. Binding of aa-Phe-tRNA^Phe^ or Ac-Phe-tRNA^Phe^ at half maximal concentrations was monitored in the presence of increasing amounts of Sdo1p. Peptidyltransferase activity was similarly monitored. Bars indicate SEM (*n* = 4 for a, b, *n* = 3 for c), *P < 0.05, **P < 0.01 (compared to wild type unless noted).

Saturation mutagenesis of the rpL10 loop revealed two classes of mutants based on their ribosome biogenesis defects. Class I mutants, typified by rpL10-S104D, were defective for subunit joining and displayed halfmer polysomes and Class II mutants, exemplified by rpL10-A106R, exhibited higher 60S/40S subunit ratios (5). While the rpL10 loop was not resolved by X-ray crystallography (8), cryo-EM studies suggested that the tip of this loop is in close proximity to the P-site tRNA (20,21). Thus, it was speculated that P-site ligand binding would be affected by these mutants. However, steady state binding of acetylated-[^14^C]Phe-tRNA^Phe^ to 80S ribosomes purified from isogenic strains expressing wild type and mutant forms of rpL10 showed no significant differences in binding of this ligand to the ribosomal P-site (Figure 2a; Supplementary Figure S1a). In contrast, the mutants showed significant changes in their ability to bind elongation ternary complex ([^14^C]Phe-tRNA^Phe^•eEF1A•GTP) to the ribosomal A-site: specifically the rpL10-S104D mutant promoted a 2-fold increase in *K*_D_ for this ligand, while the rpL10-A106R mutant promoted a 2-fold decrease (Figure 2b; Supplementary Figure S1b). To ascertain whether these differences were due to changes in affinity for the elongation factor or the aa-tRNA itself, the same experiment was performed using [^14^C]Phe-tRNA^Phe^ alone (i.e. non-enzymatic binding). Under these conditions, the aa-tRNA binding defect of the rpL10-S104D mutant was exacerbated (∼6-fold increased *K*_D_ relative to wild-type ribosomes), and this was partially ameliorated by addition of eEF1A (Figure 2b; Supplementary Figure S1d). In contrast, the binding of [^14^C]Phe-tRNA^Phe^ to wild type or rpL10-A106R ribosomes was not influenced by eEF1A. These data indicate that the tRNA binding defects are intrinsic to the ribosomal A-site and not to binding sites unique to eEF1A.

eEF2, which drives translocation, is a structural mimic of the elongation ternary complex, and includes a tRNA-like domain (Domain IV) that also interacts with the ribosomal A-site of the decoding center (22). Steady-state binding assays of purified eEF2 to 80S ribosomes, as monitored by the extent of diphtheria toxin [^14^C]-ADP ribosylation, also revealed reciprocal changes in *K*_D_ values for the two mutants (Figure 2a; Supplementary Figure S1c). Importantly, while the S104D mutant ribosomes exhibited decreased affinity for aa-tRNA and elongation ternary complex, they displayed increased affinity for eEF2. Conversely, A106R mutant ribosomes displayed increased affinity for aa-tRNA and elongation ternary complex and decreased affinity for eEF2.

Sdo1p is required at a late step in 60S maturation, coupling the GTPase activity of the Efl1p to release of Tif6p (4). Steady state binding assays using purified [^32^P]-labeled Sdo1p revealed that mutant ribosomes displayed defects in binding this ligand similar to those observed with elongation ternary complex, i.e. the rpL10-S104D mutant promoted decreased affinity for Sdo1p, while the rpL10-A106R mutant promoted increased affinity for it (Figure 2c; Supplementary Figure S1e). Sdo1p did not bind mRNA or tRNA alone, and the Sdo1pΔN mutant lacking the N-terminal FYSH domain that is essential for protein function (23) displayed negligible binding to wild-type ribosomes (Supplementary Figure S1e). Scatchard plot analyses demonstrated that Sdo1p binds to a single site on the ribosomes (Supplementary Figure S1f). Competition assays in which wild-type ribosomes were pre-incubated with increasing amounts of Sdo1p revealed that this protein stimulated binding of aa-tRNA to ribosomes in a concentration dependent manner, while inhibiting both binding of Ac-aa-tRNA to the P-site and peptidyltransferase activity (Figure 2d).

### The A106R and S104D mutants have opposing effects on the rotational equilibrium of the ribosome

During the elongation cycle, the ribosome transits through a large number of conformations, characterized at the extremes by states described as non-rotated and rotated (8,24–28). Recent single molecule experiments using *Escherichia coli* ribosomes have shown that the aa-tRNA•EF-Tu•GTP elongation ternary complex has higher affinity for non-rotated ribosomes than rotated ribosomes, and that the converse is true for EF-G (29). Thus, the ligand binding data described above suggested that the A106R and S104D mutants drive the structural equlibria of ribosomes toward either the non-rotated (substrate for binding elongation ternary complex) or rotated (substrate for eEF2) states, respectively. While chemical modification profiles and atomic resolution structures are well-defined for non-rotated and rotated *E. coli* ribosomes (24,26–28,30–34), no equivalent information exists regarding yeast ribosomes. Thus, to examine the rotational status of yeast ribosomes, it was first necessary to demonstrate that chemical protection patterns of well-defined *E. coli* and yeast ribosome complexes are similar. For *E. coli*, the standard for non-rotated ribosomes are those which are primed with polyU and contain N-Ac-PhetRNA^Phe^ in the P-site, while rotated ribosomes are primed with polyU and contain deacylated tRNA in the P/E site and EF-G-GDPNP (30). Similar complexes were prepared using yeast ribosomes (rotated yeast ribosomes contained eEF-2-GDPNP instead of EF-G-GDPNP). These two complexes, plus vacant wild-type control ribosomes were chemically probed with 1M7 (or DMSO only controls), and base reactivities were assessed by hSHAPE and quantified using ShapeFinder (19,35). Representative complete electropherograms for these three samples are shown in Supplementary Figure S2. Supplementary Table S1 shows that the chemical modification profiles of landmark *E. coli* rRNA bases are very closely matched by their yeast counterparts in the non-rotated and rotated states (compare columns 3 and 4, with columns 6 and 7). Columns 8–10 of Supplementary Table S1 also depict conversion of the statistically normalized reactivity data (19) to a scale from 0 to 4, with 4 being most reactive (18,19). Occupation of the P-site by Ac-aa-tRNA (non-rotated ribosomes) resulted in nearly identical chemical modification patterns at equivalent rRNA bases in the P-sites of the SSU and LSU, and in the LSU E-sites (indicated by color coded boxes in Supplementary Table S1). Similarly, the chemical modification patterns in the LSU P- and E-sites of rotated yeast ribosomes closely matched published data for rotated *E. coli* ribosomes. In addition, the non-rotated control was verified biochemically by comparing the ternary complex and eEF2 steady state binding profiles (Supplementary Figure S3). Note that the rotated control could not be biochemically assessed as the factor binding site was already occupied by eEF2-GDPNP. Collectively, these data demonstrate that these two complexes represent non-rotated and rotated yeast ribosomes.

**Figure 3.**
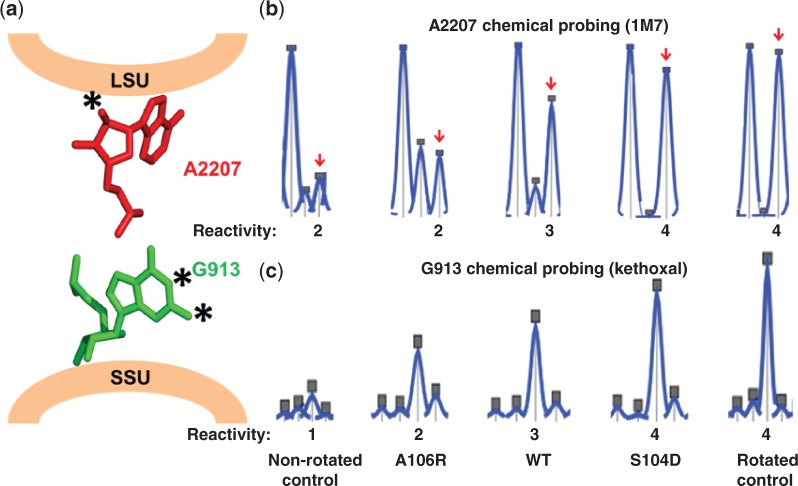
rpL10 loop mutants alter the rotational equilibrium of the ribosome. **(a)** The B7a intersubunit bridge. In the non-rotated state, A2207 (25S rRNA) forms an interaction with G913 (18S rRNA). In the rotated state, that interaction is disrupted, and the marked 2′ OH-group on A2207 becomes accessible to modification by 1M7. Similarly, marked atoms on G913 become accessible to modification by kethoxal upon rotation. **(b)** Reactivity peaks obtained by hSHAPE after probing of the landmark base A2207 (arrows) at the LSU side of the B7a intersubunit bridge with 1M7. **(c)** Reactivity peaks obtained by hSHAPE after chemical probing of the landmark base G913 at the SSU side of the B7a intersubunit bridge with kethoxal. Data were assigned five levels of reactivity: 0 (for less than a trace’s median value), 1 (for a value between the median and the mean), 2 (for a value between the mean and the first standard deviation), 3 (for a value between the first and the second standard deviation) and 4 (for values above the second standard deviation). Mean, median and standard deviation were calculated on a per trace basis.

Having established standards defining the chemical reactivity profiles of non-rotated and rotated yeast ribosomes, these were used to determine the rotational statuses of mutant ribosomes. To this end, the chemical protection profiles of ‘landmark’ basepair interactions located in several universally conserved intersubunit bridges (8,27,37–39) were examined using vacant ribosomes. Although FRET experiments revealed that vacant bacterial ribosomes are not as structurally dynamic as pre-translocation ribosomes (i.e. containing deacylated tRNA in the P site) (36), we nonetheless chose to make comparisons using vacant ribosomes for two reasons: (i) to monitor the intrinsic influence of the L10 internal loop on ribosome conformational states, i.e. in the absence of *trans*-acting factors (e.g. tRNAs) and (ii) because ribosomes harboring deacylated tRNAs in the P-sites alone do not occur in physiological conditions. The B7a intersubunit bridge undergoes dramatic rearrangements during ribosome rotation (37). Specifically, when the ribosome is in the non-rotated state, *E. coli* A702 (yeast G913) of the SSU rRNA interacts with *E. coli* A1847 (yeast A2207) of the LSU rRNA, protecting both bases from chemical attack. This interaction is disrupted when the ribosome assumes the rotated state, rendering both bases susceptible to chemical modification (Figure 3a; Supplementary Figure S4) (37). Kethoxal was used to probe G913, and 1M7 was used to probe A2207, and the extent to which these bases were modified in purified vacant isogenic wild type and mutant ribosomes, and with control rotated and non-rotated ribosomes was quantitatively assessed and normalized using hSHAPE (18). Figure 3b and c show that both of these bases were reactive along a continuum, beginning with non-rotated wild-type control (least reactive) to rpL10-A106R, vacant wild type, rpL10-S104D, and finally rotated wild-type control (most reactive). The intermediate peak heights observed with vacant wild-type ribosomes are consistent with the view that the 80S ribosome is free to transit between the two states in the absence of ligands (40). Additional key rRNA bases known to undergo structural changes during intersubunit rotation were also probed. For example, the B2a intersubunit bridge, formed between the distal loop of H69 of the LSU and h44 of the SSU is important for substrate selection on the ribosome (40), and bases comprising this bridge undergo significant rearrangement during subunit rotation, albeit not as dramatic as the B7a bridge (27). Table 1 shows that bases involved in the B2 bridge are less reactive in non-rotated control and *rpl10-*A106R mutant ribosomes than their rotated control and *rpl10*-S104D counterparts. The B3 bridge was utilized as an internal control because it is not disrupted during intersubunit rotation and is thought to be the pivot around which the subunits rotate (7,27,41). Consistent with this, no significant changes in B3 base reactivities were observed. The aa-tRNA accommodation corridor (AC) closes upon ribosome rotation, rendering the ‘gate bases’ less reactive (7,41–43). This is reflected as increased chemical reactivities of these bases (U2860, U2924 and U2926) in non-rotated control and A106R mutant ribosomes as compared to S104D and rotated controls. In addition, specific bases in the Sarcin/Ricin loop are reactive in non-rotated *E. coli* ribosomes (30). The same protection patterns are observed at the analogous yeast rRNA bases (U3023 and A3027). Collectively, the analyses shown in Table 1 support the hypothesis that the S104D mutant drives the structural equilibrium of 80S ribosomes toward the rotated state and that the A106R mutant drives it toward the non-rotated state.

**Table 1.** Establishing the rotational status of mutant ribosomes

Region	rRNA base	Non-rotated	A106R	S104D	Rotated
B7a	A2207	2	2	4	4
	G913 (SSU)	1	2	4	4
B2a	U2258	2	2	3	4
	A2262	0	0	1	2
	C1644 (SSU)	1	1	2	2
	G1645 (SSU)	0	0	2	1
B3	U2301	0	0	1	1
	G2302	0	0	0	0
	A1655 (SSU)	1	2	2	2
	U1656 (SSU)	2	2	2	2
AC	U2860	1	2	0	0
	U2924	4	2	0	1
	A2926	4	2	1	1
SRL	U3023	1	1	0	0
	A3027	2	3	0	0

Non-rotated control yeast ribosomes were primed with polyU and contained Ac-Phe-tRNA^Phe^ in the P-site. Rotated control ribosomes were primed with polyU, loaded with deacylated Phe-tRNA and incubated with eEF-2-GDPNP. These complexes, along with salt-washed vacant Rpl10-A106R and Rpl10-S104D ribosomes were chemically probed with 1M7 and analyzed by hSHAPE. B7, B2a and B3 denote the corresponding intersubunit bridges. Numerical values reflect statistically normalized reactivities on a scale of 0–4, with 4 being most reactive (18,19).

### The S104D and A106R mutants define local and long distance changes in rRNA structure corresponding to ribosome rotational status

Having established the rotational status of the mutant ribosomes, 1M7 and hSHAPE were used to quantitatively assess the chemical reactivities of approximately half (∼2000 nt) of the rRNA content of salt-washed vacant 80S ribosomes purified from isogenic cells expressing wild type and the mutant forms of rpL10. The highly reproducible quantitative data enabled subtraction analyses for each base probed. Supplementary Figure S5 employs a heat map based approach in which the reactivity data obtained from A106R mutant ribosomes were subtracted from data obtained from S104D mutant ribosomes. This analysis yields maps highlighting the differences in rRNA base reactivities between the conformational states of the two mutants. In these maps, warmer colors signify higher reactivity, and implicit higher flexibility of rRNA bases in S104D relative to A106R, while cooler colors correspond to lower reactivity, and implicit higher structure and constraint. Reactivity scale numbers denote the extent of the differences with each step in color as statistically determined (each step denoting one standard deviation) (18). These are mapped onto two-dimensional flat rRNA maps (Supplementary Figure S5a–c), onto three-dimensional (3D) atomic resolution structures (Supplementary Figure S5d and e), and are summarized in Figure 6c.

Significant differences between the two mutants were observed in the core of the PTC, the rRNA structure most proximal to the rpL10 loop (Supplementary Figure S5a). Three of the four bases proposed to play central roles in the induced fit model of peptidyltransfer (44), i.e. G2922 (*E. coli* G2553), U2924 (*E. coli* U2555) and U2954 (*E. coli* U2584) were less reactive in the rpL10-S104D mutant as compared to rpL10-A106R (Supplementary Figure S5a) suggesting a role for yeast rpL10 in this process. The universally conserved A2819 (*E. coli* A2450), G2874 (*E. coli* G2505) and U2875 (*E. coli* U2506) that constitute the ‘entrance’ to the PTC (43) also showed dramatic differences in chemical reactivities (Supplementary Figure S5a and d).

Moving outward from the loop, rpL10 is framed by Helices 38 (the A-site finger), 39, 43 and 89. Significant differences in chemical reactivities between the two mutants are seen in all these structures (Supplementary Figure S5a and b), suggesting that structural rearrangements involving the flexible loop may be transduced through the body of rpL10 to neighboring rRNA structural elements. Focusing on these elements in more detail, Helices 89 and 90–92 (Supplementary Figure S5a) form the ‘AC’ through which 3′ ends of aa-tRNAs transit as they enter the PTC (43). In this structure, U2860 in H89 (*E. coli* U2491) and U2924 in H92 (*E. coli* U2555) interact to ‘close’ the AC in the rotated state. Conversely, they do not interact in the non-rotated state and should be more extensively chemically modified when the AC is open. In support of our model, the difference map shown in Supplementary Figure S5a and summarized in Table 1 demonstrates that these bases are more protected from chemical attack in S104D mutant ribosomes as compared to A106R mutants.

rpL10 also interacts directly with Helix 39. Examination of Supplementary Figure S5b reveals large scale structural differences between the two mutant ribosomes extending from this helix to Helix 44 (PTC-distal). Importantly, these include the GTPase activating center and the H43–44 structure, upon which the P0/P1/P2 (*E. coli* L7/L12) stalk is assembled (45). This region of the LSU interacts with both the elongation ternary complex and eEF2 at different stages during the elongation cycle. As discussed below, we suggest that the structural differences identified here trace an information transmission pathway that helps the ribosome distinguish between different *trans*-acting factors at different points in its lifecycle. One such transmission pathway is shown in Supplementary Figure S5e: here, the reactivity data from Supplementary Figure S5b are plotted onto the yeast ribosome high-resolution 3D structure to illustrate a network of rRNA helices connecting the PTC-proximal and PTC-distal portions of rpL10.

The three site allosteric model posits that structural changes in the ribosomal A and E sites are linked (46), and studies from our laboratory have identified an extensive network of rRNA structural changes extending along the entire path taken by tRNAs as they transit the ribosome (7,42,47,48). Examination of the chemical modification patterns in Helices 82–88 (Supplementary Figure S5a), which trace the path of tRNAs from the A- to the E-sites in the LSU, reveals extensive differences in rRNA base reactivities between the two mutants consistent with linkage between the A- and E-sites.

The distal tip of H38 forms the LSU partner of the B1a intersubunit bridge. Although no differences in base reactivities were observed in this structure (Supplementary Figure S5b), presumably because it is intrinsically highly mobile (8,33,49,50), changes were observed in its SSU partner in the distal loop of h33, part of the 3′ major or ‘head domain’ of the SSU (Supplementary Figure S5c). A block of changes in rRNA base reactivities were also observed along the universally conserved helices 32–35 in the 3′ major domain of the 18S rRNA. These map to the mRNA entrance tunnel on the SSU opposite the decoding center (see Figure 6c). In the 3′ minor domain, A1755 (*E. coli* A1492), which plays a central role in stabilizing cognate codon:anticodon interactions in the decoding center (51), is much more protected from chemical modification in rpL10-S104D compared to rpL10-A106R ribosomes (Supplementary Figure S5c). The head of the SSU undergoes a dramatic series of rearrangements during intersubunit rotation (28,52). Multiple differences in rRNA base reactivities were also noted throughout the head domain (h38–h43), providing evidence that the rpL10 loop may influence intersubunit rotation through the B1b/c bridge in a pathway involving 5S rRNA and rpL11 (48). This is supported by pronounced differences in rRNA chemical protection patterns at the tip of H88 (Supplementary Figure S5a), which interacts with rpL11 as a monitor of P-site tRNA occupancy (47). This pathway is shown in Figure 6c.

### Identification of important points of contact through which structural changes in rpL10 are communicated to the surrounding rRNA

For structural changes in the loop of rpL10 to be broadly communicated to both subunits, information needs to be transduced through points of contact between rpL10 and local rRNA structural elements. For example, structural changes at amino acid R7, located in the N-terminal ‘hook’ of rpL10, are involved in opening and closing the AC (7). Examination of mutants identified in a previous genetic screen of *rpl10* mutants suggested two additional candidates: F94 and G81 (Supplementary Figure S9). F94 interacts with H38, and the F94I mutant was chosen for deeper analyses because of its strong resistance to anisomycin (not shown), a competitive inhibitor of aa-tRNA binding to the A-site. Replacement of the aromatic phenylalanine with isoleucine (F94I) produced ribosomes with higher affinity for elongation ternary complex and decreased affinity for eEF2, i.e. distributed toward the non-rotated state similar to the A106R mutant (Supplementary Figure S6a). hSHAPE analysis revealed that rRNA bases proximal to F94 (Supplementary Figure S6d, circled) were deprotected in the F94I mutant, presumably due to loss of interactions involving the aromatic ring of phenylalanine. Interestingly, bases further down the H38 structure (Supplementary Figure S6d, boxed) that interact with the aa-tRNA D-loop showed decreased reactivities similar to an rRNA mutant of these bases that also displayed increased affinity for elongation ternary complex (53). G81 is located at the opposite end of the body of rpL10, closer to the solvent exposed (PTC-distal) side of the protein and is one of the amino acid residues closest to the elongation factor binding site (H43). The G81D mutant was chosen because of its anisomycin hypersensitivity (not shown), and it promoted the opposite effects, i.e. decreased affinity for elongation ternary complex and increased affinity for eEF2 (Supplementary Figure S6a–c). hSHAPE analysis also revealed that rRNA bases comprising the eEF2 binding site and the GTPase-associated center in G81D underwent changes in reactivity similar to those observed with S104D (Supplementary Figure S5b). These findings suggest that, like S104D, G81D distributes ribosomes toward the rotated state.

### Effects of rpL10 loop mutants on peptidyltransferase activity and translational fidelity

**Figure 4.**
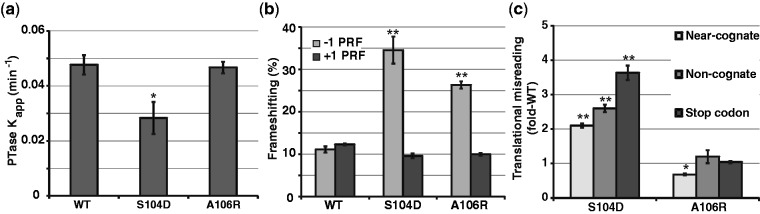
rpL10 loop mutants affect peptidyltransferase activity and translational fidelity. **(a)** Apparent rates of peptidyltransfer from single turnover peptidylpuromycin reactions for indicated ribosomes. **(b)** Programmed −1 and +1 ribosomal frameshifting values obtained using dual-luciferase reporters. **(c)** Misincorporation of a stop codon, and near- and non-cognate amino acids in mutants compared to wild-type levels as monitored using dual-luciferase reporters. Bars indicate SEM (*n* = 4), *P < 0.05, **P < 0.01.

A single round, puromycin-based assay of peptidyltransferase activity (54) revealed that S104D mutant ribosomes promoted ∼60% of peptidyltransferase activity relative to wild type and A106R mutant ribosomes (Figure 4a; Supplementary Figure S7). As peptidyltransfer occurs in the context of the non-rotated state, this further supports the hypothesis that the rpL10-S104D mutant drives the equilibrium of ribosomes toward the rotated state. Programmed −1 ribosomal frameshifting (−1 PRF) directed by L-A virus-derived sequence requires slippage of both A- and P-site tRNAs (55), while Ty*1* directed +1 PRF only requires slippage of P-site tRNA (56). Both mutants promoted enhanced −1 PRF but had no effects on +1 PRF (Figure 4b). To more completely understand how the structural changes induced by the S104D and A106R mutations impact the ability of the ribosome to accurately decode mRNAs, we employed dual-luciferase translational fidelity assays designed to measure rates of misreading of near- and non-cognate tRNAs (11), and misreading of a UAA termination codon (10). The S104D mutant was generally more inaccurate than wild-type, the magnitude of which became greater as the extent of codon/anticodon mismatches increased (Figure 4c). In contrast, decoding was largely unaffected in cells expressing the A106R mutant, although decoding of near-cognate tRNAs was slightly more accurate.

### An intra-ribosomal suppressor of the S104D mutant reveals the importance of LSU allostery in function of the mature ribosome

**Figure 5.**
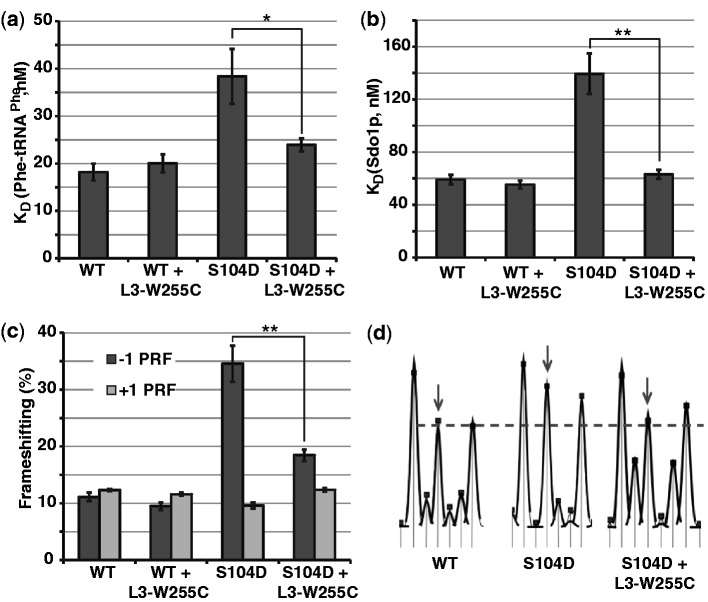
A mutant of rpL10 can be intrinsically suppressed with a mutant of rpL3. **(a** and **b)** Binding aa-tRNA and Sdo1p to indicated ribosomes. **(c)** Frameshifting analyses. **(d)** Structural probing of the landmark base A2207 (arrows) at the LSU side of the B7a intersubunit bridge with 1M7. Bars indicate SEM (*n* = 4 for a and c, *n* = 3 for b), *P < 0.05, **P < 0.01.

**Figure 6.**
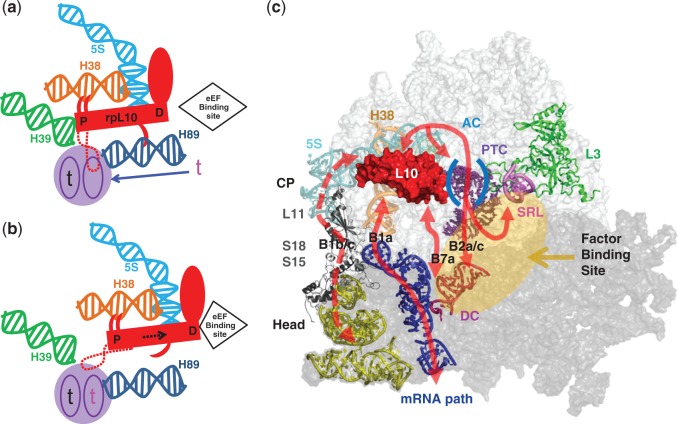
Models of rpL10 function: rpL10 is at the center of a cascade of allosteric communication pathways throughout the ribosome. **(a)** The rpL10 loop ‘flipped in’ conformation is the substrate for aa-tRNA and Sdo1p. P, PTC-proximal; D, PTC-distal. **(b)** The ‘flipped out’ loop conformation, substrate for eEF2. Binding of an aa-tRNA (indicated by the red ‘t’) causes displacement of the loop from the A-site, precipitating structural rearrangements in rpL10. These include lateral displacement of the main body of the protein (dashed black arrow) and H38 toward the elongation factor binding site, creating the binding platform for eEF2. Release of the N-terminal hook of rpL10 from H89 enables closing of the aa-tRNA AC. These movements also initiate allosteric transmission of information through the communication pathways shown in (c) to distantly located functional centers of the ribosome to set the stage for the next phase of elongation. These include rearrangements in the E-site in preparation for release of deacylated tRNA, and interactions with the decoding center and SSU to initiate subunit rotation. **(c)** Summary of chemical probing experiments mapping the allosteric information exchange pathways emanating from rpL10 to all the functional centers of the ribosome to influence intersubunit rotation. Intersubunit bridges B1a, B1b/c B2a/c and B7a and ribosomal proteins L3, L10, L11, S15 and L18 are labeled. CP, central protuberance of the LSU; SRL, Sarcin/Ricin loop; DC, decoding center.

If the rpL10 mutants affect the distribution of ribosomes between the classical and rotated states by altering the distribution of allosteric structures intrinsic to the ribosome, then mutants of other ribosomal components that promote opposing effects may be able to suppress the defects exhibited by the *rpL10* mutants. The L3-W255C mutant, which lies in a flexible loop (the W-finger) on the opposite side of the aa-tRNA AC from rpL10 (Figure 6c) promotes increased affinity of aa-tRNA to the A-site and decreased affinity for eEF2 (54). A high copy plasmid expressing L3-W255C was able to almost completely suppress the biochemical (binding of aa-tRNA and Sdo1p to the A-site), and translational fidelity (−1 PRF) defects of the rpL10-S104D mutant (Figure 5a–c; Supplementary Figure S8c). The ability of this mutant to correct the changes in rRNA structure at the B7a bridge promoted by the rpL10-S104D mutant (Figure 5d) demonstrates that the proper rotational equilibrium was re-established. The control experiment, co-expression of L3-W255C with the wild-type *RPL3*, did not affect any of these parameters. Interestingly, this mutant did not suppress the rpL10-S104D slow growth defect (data not shown) or the ribosome biogenesis defect of rpl10-S104D, as assessed by polysome profiling (Supplementary Figure S8). No mutants of L3 able to suppress the rpL10-A106R mutant were identified.

## DISCUSSION

Eukaryotic rpL10 and its prokaryotic homologs L16 in bacteria and L10e in *archaea* contain a conserved internal loop that approaches the catalytic center of the LSU. This loop is not resolved in atomic resolution structures of vacant yeast (8) or prokaryotic ribosomes (57), suggesting that this structure is dynamic. However, the internal loop is resolved in high-resolution crystal structures of bacterial ribosomes containing P-site tRNA (20,58) and in cryo-EM imaging of translating yeast ribosomes (59), suggesting that it is stabilized by tRNA binding. While the loop is shorter in the bacterial and archaeal homologs, the N-terminus of the L27 proteins from these kingdoms appear to provide the structural mimic for the tip of this loop (60). Here, we have shown that mutations of this loop affect the rotational status of the ribosome, altering the ribosome’s affinity for Sdo1p at the P-site, for aa-tRNAs and eEF2 at the A-site and globally affecting ribosome function from biogenesis through translational fidelity.

Current models posit that ribosomal rotational status is solely determined by binding of different tRNA species and trans-acting GTPases (26–28,61). However, the findings presented here suggest that control of rotation is an intrinsic property of the ribosome. We propose that when the A-site is unoccupied by ligand, the flexible rpL10 loop can sample this space: we call this the ‘flipped in’ conformation. In contrast, occupation of the A-site by tRNAs displaces, or ‘flips out’ this loop (Figure 6a and b). The effects of the two rpL10 mutants on A-site associated functions and on ribosome structure in the absence of ligands suggest that this loop has an intrinsic, central role in establishing the rotational status of the ribosome. The finding that non-enzymatic binding of aa-tRNA to the A-site is exacerbated and that peptidyltransferase activity is diminished in rpL10-S104D provide additional support for this idea. Addition of the positively charged arginine residue may create new charge–charge interactions with the A-site to stabilize the ‘flipped in’ state making it more difficult to displace by aa-tRNA (Figure 6a). Conversely, addition of a negatively charged residue in the S104D mutation may interfere with the ability of the loop to stably occupy the A-site due to charge repulsion effects with this rRNA-rich environment, thus stabilizing the ‘flipped out’ conformation (Figure 6b).

How can a change in the position of a small peptide loop affect the conformation of a large and complex macromolecule like the ribosome? We suggest that ligand occupancy information is first transduced locally by lateral movement of the body of rpL10 as a result of dynamic changes in loop positioning. This information is allosterically transduced to more distant regions of the ribosome through rRNA networks thus setting the ribosome rotational mechanism in motion. In this model, the body of rpL10 functions akin to a piston that is physically linked to multiple functional centers of the machine (visualized in Figure 6, primary hSHAPE data summarized in Supplementary Figure S9). The allosteric information pathways identified by hSHAPE analysis frame the path through which tRNA moves through the ribosome during translation, including the AC and decoding center, the PTC and the E-site. They also encompass the critical intersubunit bridges involved in rotation and the elongation factor binding site. Previous studies identified other flexible peptide elements that are used by the ribosome as allosteric switches. These include the N-terminal hook of rpL10 (7), the W-finger, N-terminal extension, and Basic Thumb elements of rpL3 (14,54,62), an internal loop located in rpL5 (63) and the P-site loop of rpL11 (47). Importantly, there is a considerable degree of overlap between the allosteric networks identified in these and other studies (41,42,48,53,64,65), indicating that they are all components a single network of ‘switches’ and ‘wires’ that coordinate ribosome structure with function. This is further supported by the observation in the current study that a mutant in one switch, L3-W255C, can suppress the biochemical and translational fidelity defects of a second, i.e. the rpL10-S104D loop mutant. What distinguishes the rpL10 loop from other elements is that it is a primary sensor of the arrival of ligands at the A-site. As a result, defects in this initial step are amplified through a series of outwardly radiating allosteric networks, broadly impacting ribosome biogenesis, elongation and termination. It should be noted that while we interpret our results as evidence for the influence of rpL10 on ribosome equilibria, alternative explanations are also possible. For example, these results could be interpreted as evidence for changes in rRNA folding and the structure of the LSU. However, incorporation of L10 occurs very late in the process of eukaryotic ribosome biogenesis after the pre-60S subunit has been exported to the cytoplasm, well after the core of the LSU has been folded. Thus, the effects of these single amino acid mutants of rpL10 on rRNA folding are likely to be minimal.

The rotational status of the 80S ribosome is an important determinant for binding of *trans*-acting factors, optimizing PTC activity and translational fidelity. Both mutants promoted increased rates of −1 PRF (Figure 4b), which requires slippage of both A- and P-site tRNAs (66), but did not affect +1 PRF, which only requires slippage of P-site tRNA (56). This is consistent with the observation that the rpL10 loop mutants only affected A-site tRNA binding. Increased utilization of near- and non-cognate tRNAs, and termination codon readthrough by the rpL10-S104D mutant (Figure 4c) may be explained by the observation that A1755 (*E. coli* A1492) in h44 was hyperprotected from chemical modification (Supplementary Figure S5c). Flexibility of this base is critical for accurate decoding by stabilizing a mini-helix between cognate codon:anticodon interactions (67). Concurrently, LSU A2256 (*E. coli* A1913) in H69 was hyper-reactive in rpL10-S104D ribosomes (Supplementary Figure S5a). This base also plays a central role in mRNA decoding where it is paired with A1492 in the non-rotated state, but is unpaired and flexible in the rotated state (68). We suggest that the propensity of rpL10-S104D ribosomes to assume the rotated stated limits the ability of these bases to participate in mini-helix formation, leading to increased utilization of non-cognate ligands.

**Figure 7.**
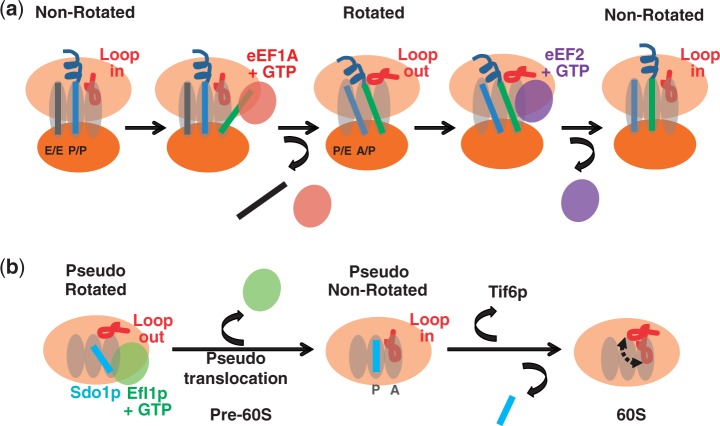
Involvement of the rpL10 loop in ribosome rotation throughout the ribosomal life cycle. **(a)** The elongation cycle of translation: rpL10 loop positioning and ligand binding. The elongation cycle begins at left with the ribosome in the non-rotated state where the E-site contains a deacylated tRNA, the P-site is occupied by peptidyl tRNA, and the un-occupied A-site can be sampled by the rpL10 loop, i.e. the ‘flipped in’ conformation. Following elongation ternary complex binding, aa-tRNA accommodation and peptidyltransfer, tRNAs assume the hybrid states and the loop assumes the ‘flipped out’ conformation, signaling the ribosome to assume the rotated state. eEF2 binds to rotated ribosomes, resulting in translocation, and the elongation cycle begins anew. **(b)** The ‘test drive’. Sdo1p and Efl1p interact with the pre-60S subunit in the pseudo-rotated state. Efl1p-mediated pseudo-translocation drives Sdo1p into the P-site, stabilizing the pseudo-non-rotated state with the rpL10 loop in the ‘flipped in’ conformation. This is followed by release of the anti-association factor Tif6p and Sdo1p, promoting the final steps of 60S maturation.

Considering the mechanisms in place to ensure fidelity of gene expression we expect that safeguards have evolved to ensure that only correctly functioning ribosomes are utilized in translation. Ribosome assembly culminates in cytoplasmic maturation where essential ribosomal proteins are added, and transacting factors are released (69). A critical step in this pathway is the release of the anti-association factor Tif6p by the concerted action of Sdo1p and the eEF2 paralog Efl1p (70,71). The observation that suppressing mutations in Efl1p appear to facilitate a conformational change in the protein akin to the conformational change observed in eEF2 engendered the proposal that the LSU undergoes a ‘test drive’ in which the integrity of the P-site is assessed through activation of the GTPase of Efl1p (5). Notably, similar functional checkpoints governing pre-40S maturation have recently been proposed (72,73). The findings presented in the current study add further support and detail to this model. Specifically, the ability of Sdo1p to compete for Ac-aa-tRNA binding in the P-site, inhibit peptidyltransferase activity and stimulate aa-tRNA binding to the A-site [similar to the stimulatory effect of P-site bound Ac-aa-tRNA on aa-tRNA binding to the A-site (74)] all suggest that Sdo1p interacts with the P-site, stabilizing a pseudo-non-rotated state of the LSU. The structure of Sdo1p has been likened to tRNA (75), and we propose that Sdo1p is a mimic for a P-site ligand that couples the GTPase activity of the elongation factor mimic Efl1p (4) to drive a pseudo-translocation event on the LSU (Figure 7). Because Efl1p and Sdo1p appear to act on 60S independent of 40S, we suggest that the rpL10-S104D and -A106R mutants promote conformational changes within the 60S subunit alone that are analogous to those in the 60S subunit in the context of rotated and non-rotated 80S ribosomes. The 60S subunit ‘test drive’ appears to provide the primary quality control check on assembly and function of the LSU before it is released into the pool of actively translating ribosomes. Sdo1p binding defects in the ‘pseudo-rotated’ S104D mutant can be intrinsically suppressed with the rpL3-W255C mutant, likely due to its ability to re-establish the correct pre-LSU conformation. However, the failure of this mutant to rescue the ribosome biogenesis defect suggests that quality control involves more than mere monitoring of ligand binding.

The biomedical importance of the rpL10 loop was recently highlighted by the discovery that a significant fraction of T-cell acute lymphoblastic leukemia patients harbor mutations of R98, and in one patient at Q123, both of which lie at the base of the loop (76). Remarkably, these mutations prevent the release of Tif6p and Nmd3p, but can be suppressed by mutations in Nmd3p, indicating that like the S104D mutation, R98 and Q123 mutations in human *Rpl10* cause a failure during the ‘test drive’ of the 60S subunit. These findings suggest that defects in ribosome biogenesis and/or in translational fidelity may be drivers of this neoplastic disease. Investigations are currently underway in our laboratories to address these questions.

## SUPPLEMENTARY DATA

Supplementary Data are available at NAR Online.

## FUNDING

Public Health Service [2 R01 GM058859-11 and 3 R01 GM053655-15S1 to J.D.D., 2 RO1 GM53655 and 3 R01 GM053655-15S1 to A.W.J.]; Partially supported by NIH training [T32GM080201 to S.O.S.]. Funding for open access charge: The Public Health Service [R01 GM053655-15S1, R01 GM058859, RO1 GM53655 and T32GM080201].

*Conflict of interest statement*. None declared.

## References

[1] Jenner L, Melnikov S, de Loubresse NG, Ben Shem A, Iskakova M, Urzhumtsev A, Meskauskas A, Dinman J, Yusupova G, Yusupov M (2012). Crystal structure of the 80S yeast ribosome. Curr. Opin. Struct. Biol..

[2] West M, Hedges JB, Chen A, Johnson AW (2005). Defining the order in which Nmd3p and Rpl10p load onto nascent 60S ribosomal subunits. Mol. Cell Biol..

[3] Menne TF, Goyenechea B, Sanchez-Puig N, Wong CC, Tonkin LM, Ancliff PJ, Brost RL, Costanzo M, Boone C, Warren AJ (2007). The Shwachman-Bodian-Diamond syndrome protein mediates translational activation of ribosomes in yeast. Nat. Genet..

[4] Finch AJ, Hilcenko C, Basse N, Drynan LF, Goyenechea B, Menne TF, Gonzalez FA, Simpson P, D'Santos CS, Arends MJ (2011). Uncoupling of GTP hydrolysis from eIF6 release on the ribosome causes Shwachman-Diamond syndrome. Genes Dev..

[5] Bussiere C, Hashem Y, Arora S, Frank J, Johnson AW (2012). Integrity of the P-site is probed during maturation of the 60S ribosomal subunit. J. Cell Biol..

[6] Hedges J, West M, Johnson AW (2005). Release of the export adapter, Nmd3p, from the 60S ribosomal subunit requires Rpl10p and the cytoplasmic GTPase Lsg1p. EMBO J..

[7] Petrov AN, Meskauskas A, Roshwalb SC, Dinman JD (2008). Yeast ribosomal protein L10 helps coordinate tRNA movement through the large subunit. Nucleic Acids Res..

[8] Ben Shem A, Garreau dL, Melnikov S, Jenner L, Yusupova G, Yusupov M (2011). The structure of the eukaryotic ribosome at 3.0 A resolution. Science.

[9] Hofer A, Bussiere C, Johnson AW (2007). Mutational analysis of the ribosomal protein RPL10 from yeast. J. Biol. Chem..

[10] Harger JW, Dinman JD (2003). An *in vivo* dual-luciferase assay system for studying translational recoding in the yeast *Saccharomyces cerevisiae*. RNA.

[11] Plant EP, Nguyen P, Russ JR, Pittman YR, Nguyen T, Quesinberry JT, Kinzy TG, Dinman JD (2007). Differentiating between near- and non-cognate codons in *Saccharomyces cerevisiae*. PLoS One.

[12] Jacobs JL, Dinman JD (2004). Systematic analysis of bicistronic reporter assay data. Nucleic Acids Res..

[13] Leshin JA, Rakauskaite R, Dinman JD, Meskauskas A (2010). Enhanced purity, activity and structural integrity of yeast ribosomes purified using a general chromatographic method. RNA Biol..

[14] Meskauskas A, Dinman JD (2010). A molecular clamp ensures allosteric coordination of peptidyltransfer and ligand binding to the ribosomal A-site. Nucleic Acids Res..

[15] Meskauskas A, Petrov AN, Dinman JD (2005). Identification of functionally important amino acids of ribosomal protein L3 by saturation mutagenesis. Mol. Cell Biol..

[16] Ortiz PA, Ulloque R, Kihara GK, Zheng H, Kinzy TG (2006). Translation elongation factor 2 anticodon mimicry domain mutants affect fidelity and diphtheria toxin resistance. J. Biol. Chem..

[17] Wilkinson KA, Merino EJ, Weeks KM (2006). Selective 2′-hydroxyl acylation analyzed by primer extension (SHAPE): quantitative RNA structure analysis at single nucleotide resolution. Nat. Protoc..

[18] Leshin JA, Heselpoth R, Belew AT, Dinman JD (2011). High throughput structural analysis of yeast ribosomes using hSHAPE. RNA Biol..

[19] Vasa SM, Guex N, Wilkinson KA, Weeks KM, Giddings MC (2008). ShapeFinder: a software system for high-throughput quantitative analysis of nucleic acid reactivity information resolved by capillary electrophoresis. RNA.

[20] Voorhees RM, Weixlbaumer A, Loakes D, Kelley AC, Ramakrishnan V (2009). Insights into substrate stabilization from snapshots of the peptidyl transferase center of the intact 70S ribosome. Nat. Struct. Mol. Biol..

[21] Armache JP, Jarasch A, Anger AM, Villa E, Becker T, Bhushan S, Jossinet F, Habeck M, Dindar G, Franckenberg S (2010). Localization of eukaryote-specific ribosomal proteins in a 5.5-A cryo-EM map of the 80S eukaryotic ribosome. Proc. Natl Acad. Sci. U.S.A..

[22] Spahn CM, Gomez-Lorenzo MG, Grassucci RA, Jorgensen R, Andersen GR, Beckmann R, Penczek PA, Ballesta JP, Frank J (2004). Domain movements of elongation factor eEF2 and the eukaryotic 80S ribosome facilitate tRNA translocation. EMBO J..

[23] Shammas C, Menne TF, Hilcenko C, Michell SR, Goyenechea B, Boocock GR, Durie PR, Rommens JM, Warren AJ (2005). Structural and mutational analysis of the SBDS protein family. Insight into the leukemia-associated Shwachman-Diamond Syndrome. J. Biol. Chem..

[24] Frank J, Agrawal RK (2000). A ratchet-like inter-subunit reorganization of the ribosome during translocation. Nature.

[25] Valle M, Zavialov A, Sengupta J, Rawat U, Ehrenberg M, Frank J (2003). Locking and unlocking of ribosomal motions. Cell.

[26] Fischer N, Konevega AL, Wintermeyer W, Rodnina MV, Stark H (2010). Ribosome dynamics and tRNA movement by time-resolved electron cryomicroscopy. Nature.

[27] Zhang W, Dunkle JA, Cate JH (2009). Structures of the ribosome in intermediate states of ratcheting. Science.

[28] Dunkle JA, Wang L, Feldman MB, Pulk A, Chen VB, Kapral GJ, Noeske J, Richardson JS, Blanchard SC, Cate JH (2011). Structures of the bacterial ribosome in classical and hybrid states of tRNA binding. Science.

[29] Chen J, Petrov A, Tsai A, O'Leary SE, Puglisi JD (2013). Coordinated conformational and compositional dynamics drive ribosome translocation. Nat. Struct. Mol. Biol..

[30] Moazed D, Noller HF (1989). Intermediate states in the movement of transfer RNA in the ribosome. Nature.

[31] Stern S, Moazed D, Noller HF (1988). Structural analysis of RNA using chemical and enzymatic probing monitored by primer extension. Methods Enzymol..

[32] Agrawal RK, Spahn CM, Penczek P, Grassucci RA, Nierhaus KH, Frank J (2000). Visualization of tRNA movements on the *Escherichia coli* 70S ribosome during the elongation cycle. J. Cell Biol..

[33] Cate JH, Yusupov MM, Yusupova GZ, Earnest TN, Noller HF (1999). X-ray crystal structures of 70S ribosome functional complexes. Science.

[34] Gao H, Sengupta J, Valle M, Korostelev A, Eswar N, Stagg SM, Van Roey P, Agrawal RK, Harvey SC, Sali A (2003). Study of the structural dynamics of the *E. coli* 70S ribosome using real-space refinement. Cell.

[35] Merino EJ, Wilkinson KA, Coughlan JL, Weeks KM (2005). RNA structure analysis at single nucleotide resolution by selective 2′-hydroxyl acylation and primer extension (SHAPE). J. Am. Chem. Soc..

[36] Cornish PV, Ermolenko DN, Noller HF, Ha T (2008). Spontaneous intersubunit rotation in single ribosomes. Mol. Cell.

[37] Taylor DJ, Devkota B, Huang AD, Topf M, Narayanan E, Sali A, Harvey SC, Frank J (2009). Comprehensive molecular structure of the eukaryotic ribosome. Structure.

[38] Ben Shem A, Jenner L, Yusupova G, Yusupov M (2010). Crystal structure of the eukaryotic ribosome. Science.

[39] Schmeing TM, Ramakrishnan V (2009). What recent ribosome structures have revealed about the mechanism of translation. Nature.

[40] Fischer N, Konevega AL, Wintermeyer W, Rodnina MV, Stark H (2010). Ribosome dynamics and tRNA movement by time-resolved electron cryomicroscopy. Nature.

[41] Rakauskaite R, Dinman JD (2011). Mutations of highly conserved bases in the peptidyltransferase center induce compensatory rearrangements in yeast ribosomes. RNA.

[42] Rakauskaite R, Dinman JD (2008). rRNA mutants in the yeast peptidyltransferase center reveal allosteric information networks and mechanisms of drug resistance. Nucleic Acids Res..

[43] Sanbonmatsu KY, Joseph S, Tung CS (2005). Simulating movement of tRNA into the ribosome during decoding. Proc. Natl Acad. Sci. U.S.A..

[44] Schmeing TM, Huang KS, Strobel SA, Steitz TA (2005). An induced-fit mechanism to promote peptide bond formation and exclude hydrolysis of peptidyl-tRNA. Nature.

[45] Remacha M, Jimenez-Diaz A, Santos C, Briones E, Zambrano R, Rodriguez Gabriel MA, Guarinos E, Ballesta JP (1995). Proteins P1, P2, and P0, components of the eukaryotic ribosome stalk. New structural and functional aspects. Biochem. Cell Biol..

[46] Nierhaus KH (1990). The allosteric three-site model for the ribosomal elongation cycle: features and future. Biochemistry.

[47] Rhodin MH, Dinman JD (2010). A flexible loop in yeast ribosomal protein L11 coordinates P-site tRNA binding. Nucleic Acids Res..

[48] Rhodin MH, Dinman JD (2011). An extensive network of information flow through the B1b/c intersubunit bridge of the yeast ribosome. PLoS ONE.

[49] Noller HF, Yusupov MM, Yusupova GZ, Baucom A, Lieberman K, Lancaster L, Dallas A, Fredrick K, Earnest TN, Cate JH (2001). Structure of the ribosome at 5.5 A resolution and its interactions with functional ligands. Cold Spring Harb. Symp. Quant. Biol..

[50] Selmer M, Dunham CM, Murphy FV, Weixlbaumer A, Petry S, Kelley AC, Weir JR, Ramakrishnan V (2006). Structure of the 70S ribosome complexed with mRNA and tRNA. Science.

[51] Ogle JM, Brodersen DE, Clemons WM, Tarry MJ, Carter AP, Ramakrishnan V (2001). Recognition of cognate transfer RNA by the 30S ribosomal subunit. Science.

[52] Ratje AH, Loerke J, Mikolajka A, Brunner M, Hildebrand PW, Starosta AL, Donhofer A, Connell SR, Fucini P, Mielke T (2010). Head swivel on the ribosome facilitates translocation by means of intra-subunit tRNA hybrid sites. Nature.

[53] Rakauskaite R, Dinman JD (2006). An arc of unpaired “hinge bases” facilitates information exchange among functional centers of the ribosome. Mol. Cell Biol..

[54] Meskauskas A, Dinman JD (2007). Ribosomal protein L3: Gatekeeper to the A-site. Mol. Cell.

[55] Dinman JD (2012). Mechanisms and implications of programmed translational frameshifting. Wiley Interdiscip. Rev. RNA.

[56] Belcourt MF, Farabaugh PJ (1990). Ribosomal frameshifting in the yeast retrotransposon Ty: tRNAs induce slippage on a 7 nucleotide minimal site. Cell.

[57] Kavran JM, Steitz TA (2007). Structure of the base of the L7/L12 stalk of the *Haloarcula marismortui* large ribosomal subunit: analysis of L11 movements. J. Mol. Biol..

[58] Korostelev A, Trakhanov S, Laurberg M, Noller HF (2006). Crystal structure of a 70S ribosome-tRNA complex reveals functional interactions and rearrangements. Cell.

[59] Armache JP, Jarasch A, Anger AM, Villa E, Becker T, Bhushan S, Jossinet F, Habeck M, Dindar G, Franckenberg S (2010). Cryo-EM structure and rRNA model of a translating eukaryotic 80S ribosome at 5.5-A resolution. Proc. Natl Acad. Sci. U.S.A..

[60] Gao YG, Selmer M, Dunham CM, Weixlbaumer A, Kelley AC, Ramakrishnan V (2009). The structure of the ribosome with elongation factor G trapped in the posttranslocational state. Science.

[61] Dunkle JA, Cate JH (2010). Ribosome structure and dynamics during translocation and termination. Annu. Rev. Biophys..

[62] Meskauskas A, Dinman JD (2008). Ribosomal protein L3 functions as a ‘rocker switch' to aid in coordinating of large subunit-associated functions in eukaryotes and Archaea. Nucleic Acids Res..

[63] Meskauskas A, Dinman JD (2001). Ribosomal protein L5 helps anchor peptidyl-tRNA to the P-site in *Saccharomyces cerevisiae*. RNA.

[64] Kiparisov S, Petrov A, Meskauskas A, Sergiev PV, Dontsova OA, Dinman JD (2005). Structural and functional analysis of 5S rRNA. Mol. Genet. Genomics.

[65] Meskauskas A, Russ JR, Dinman JD (2008). Structure/function analysis of yeast ribosomal protein L2. Nucleic Acids Res..

[66] Jacks T, Madhani HD, Masiraz FR, Varmus HE (1988). Signals for ribosomal frameshifting in the Rous Sarcoma Virus gag-pol region. Cell.

[67] Carter AP, Clemons WM, Brodersen DE, Morgan-Warren RJ, Wimberly BT, Ramakrishnan V (2000). Functional insights from the structure of the 30S ribosomal subunit and its interactions with antibiotics. Nature.

[68] Demeshkina N, Jenner L, Westhof E, Yusupov M, Yusupova G (2012). A new understanding of the decoding principle on the ribosome. Nature.

[69] Lo KY, Li Z, Bussiere C, Bresson S, Marcotte EM, Johnson AW (2010). Defining the pathway of cytoplasmic maturation of the 60S ribosomal subunit. Mol. Cell.

[70] Valenzuela DM, Chaudhuri A, Maitra U (1982). Eukaryotic ribosomal subunit anti-association activity of calf liver is contained in a single polypeptide chain protein of Mr = 25,500 (eukaryotic initiation factor 6). J. Biol. Chem..

[71] Russell DW, Spremulli LL (1979). Purification and characterization of a ribosome dissociation factor (eukaryotic initiation factor 6) from wheat germ. J. Biol. Chem..

[72] Strunk BS, Novak MN, Young CL, Karbstein K (2012). A translation-like cycle is a quality control checkpoint for maturing 40S ribosome subunits. Cell.

[73] Lebaron S, Schneider C, van Nues RW, Swiatkowska A, Walsh D, Bottcher B, Granneman S, Watkins NJ, Tollervey D (2012). Proofreading of pre-40S ribosome maturation by a translation initiation factor and 60S subunits. Nat. Struct. Mol. Biol..

[74] Rheinberger HJ, Sternbach H, Nierhaus KH (1981). Three tRNA binding sites on *Escherichia coli* ribosomes. Proc. Natl Acad. Sci. U.S.A..

[75] Ng CL, Waterman DG, Koonin EV, Walters AD, Chong JP, Isupov MN, Lebedev AA, Bunka DH, Stockley PG, Ortiz-Lombardia M (2009). Conformational flexibility and molecular interactions of an archaeal homologue of the Shwachman-Bodian-Diamond syndrome protein. BMC Struct. Biol..

[76] De Keersmaecker K, Atak ZK, Li N, Vicente C, Patchett S, Girardi T, Gianfelici V, Geerdens E, Clappier E, Porcu M (2013). Exome sequencing identifies mutation in CNOT3 and ribosomal genes RPL5 and RPL10 in T-cell acute lymphoblastic leukemia. Nat. Genet..

